# Transcriptomic responses of *Solanum tuberosum* cv. Pirol to arbuscular mycorrhiza and potato virus Y (PVY) infection

**DOI:** 10.1007/s11103-024-01519-9

**Published:** 2024-11-11

**Authors:** Edyta Deja-Sikora, Marcin Gołębiewski, Katarzyna Hrynkiewicz

**Affiliations:** 1 Department of Microbiology, Faculty of Biological and Veterinary Sciences, Nicolaus Copernicus University, Lwowska 1, 87-100 Torun, Poland; 2 Department of Plant Physiology and Biotechnology, Faculty of Biological and Veterinary Sciences, Nicolaus Copernicus University, Lwowska 1, 87-100 Torun, Poland; 3 Centre for Modern Interdisciplinary Technologies, Nicolaus Copernicus University, Wilenska 4, 87-100 Torun, Poland

**Keywords:** *Solanum tuberosum*, Potato virus Y (PVY), Arbuscular mycorrhizal fungi (AMF), Transcriptomic analysis, Pathogenesis-related proteins (PR), Plant defense response

## Abstract

Arbuscular mycorrhizal fungi (AMF) serve as both plant symbionts and allies in resisting pathogens and environmental stresses. Mycorrhizal colonization of plant roots can influence the outcomes of plant-pathogen interactions by enhancing specific host defense mechanisms. The transcriptional responses induced by AMF in virus-infected plants remain largely unexplored. In the presented study, we employed a comprehensive transcriptomic approach and qPCR to investigate the molecular determinants underlying the interaction between AMF and potato virus Y (PVY) in *Solanum tuberosum* L. Our primary goal was to identify the symbiosis- and defense-related determinants activated in mycorrhizal potatoes facing PVY. Through a comparative analysis of mRNA transcriptomes in experimental treatments comprising healthy and PVY-infected potatoes colonized by two AMF species, *Rhizophagus regularis* or *Funneliformis mosseae*, we unveiled the overexpression of genes associated with mycorrhiza, including nutrient exchange, lipid transfer, and cell wall remodeling. Furthermore, we identified several differentially expressed genes upregulated in all mycorrhizal treatments that encoded pathogenesis-related proteins involved in plant immune responses, thus verifying the bioprotective role of AMF. We investigated the relationship between mycorrhiza levels and PVY levels in potato leaves and roots. We found accumulation of the virus in the leaves of mycorrhizal plants, but our studies additionally showed a reduced PVY content in potato roots colonized by AMF, which has not been previously demonstrated. Furthermore, we observed that a virus-dependent reduction in nutrient exchange could occur in mycorrhizal roots in the presence of PVY. These findings provide an insights into the interplay between virus and AMF.

**Supplementary Information:**

The online version contains supplementary material available at 10.1007/s11103-024-01519-9.

## Key message

The article, based on the results of transcriptomic analysis, identifies the symbiosis-related and defense-related determinants activated in mycorrhizal potatoes infected by potato virus Y (PVY).

**Supplementary Information:**

The online version contains supplementary material available at 10.1007/s11103-024-01519-9.

## Introduction

Potato (*Solanum tuberosum* L. belonging to the Solanaceae family), one of a crucial non-grain crops globally, faces numerous pathogens, much like other cultivated plants. A significant threat to potato yields are phytoviruses, which are the cause of up to 50% of plant diseases (Whitfield et al. 2015). Among these viruses, potato virus Y (PVY) stands out as a major pathogen, negatively impacting tuber quantity and quality (Weber et al. 2021). PVY regularly devastates the potato plantations worldwide, mainly due to its high prevalence and fast transmission involving insect vectors, i.e., aphids. Enhancing natural plant defense mechanisms against viruses becomes urgent in order to mitigate yield losses, as it represents the most effective and dependable strategy for safeguarding plants. It is well documented that the beneficial plant-associated microorganisms, can play the critical role in maintenance of plant health and environmental fitness. These microbial allies are able to alleviate the plant disease development, as they modulate the host responses to biotic stressors, thus shaping the outcome of plant-pathogen interaction (Deja-Sikora et al. 2020). Arbuscular mycorrhizal fungi (AMF) belong to the symbiotic fungi known for their bioprotective capabilities, hence they are increasingly considered to be useful in pathogen biocontrol (Weng et al. 2022). These fungi are ubiquitous soil inhabitants forming the widespread mutualistic association called arbuscular mycorrhiza (AM) with roots of numerous plant species, including many important crops (Zhu et al. 2022). In agriculture, AMF are recognized as pivotal providers of ecosystem services (Chen et al. 2018). Their positive impact on plant nutrition and fitness was extensively documented, encompassing enhanced uptake of phosphorus and nitrogen-containing minerals, as well as increased resilience to biotic and abiotic stresses (Jung et al. 2012; Lenoir et al. 2016). Additionally, numerous reports indicated that AMF have the ability to reduce the occurrence and severity of plant diseases caused by various pathogens, e.g. *Fusarium oxysporum*, *Blumeria graminis*, *Alternaria alternata*, *Alternaria solani*, and *Rhizoctonia solani* (Song et al. 2015; Nair et al. 2015; Mustafa et al. 2017; Wu et al. 2021; Pu et al. 2022). Similarly, several studies indicated the beneficial effect of AMF on the course of virus-induced diseases, encompassing e.g., the mitigation of oxidative stress caused by PVY in potato or attenuation of TYLCSV (tomato yellow leaf curl Sardinia virus) symptoms in tomato (Maffei et al. 2014; Deja-Sikora et al. 2020). Although many investigations attempted to explain the mechanisms of AMF-dependent plant tolerance or even resistance to invaders (Cameron et al. 2013; Schouteden et al. 2015; Wang et al. 2022), the molecular determinants involved in these multilateral interactions remain incompletely recognized. A growing number of studies report the reprogramming of defense gene expression patterns after the mycorrhiza establishment. This phenomenon known as priming is suggested to underlie a faster and more effective activation of plant defense mechanisms when faced with a pathogen attack (Jung et al. 2012; Hao et al. 2019; Goddard et al. 2021; Stratton et al. 2022). Based on current knowledge, it is hypothesized that the colonization of plant roots by AMF results in mycorrhiza-induced resistance (MIR) through the activation of MAMP-triggered immunity (MTI) (Kadam et al. 2020). Numerous studies have indicated that the efficacy of MIR is associated with the increased level of endogenous jasmonic acid (JA), a key regulator in plant defense, and rapid activation of the JA-dependent signaling pathway (Pozo and Azcón-Aguilar 2007; Nair et al. 2015; Jiang et al. 2021; Zhang et al. 2022). The other reports have highlighted that the combination of increased plant nutrition and enhanced defense is a key factor in improving the performance of mycorrhizal plants, especially when they are affected by pests (Fiorilli et al. 2018; Zhang et al. 2022; Stratton et al. 2022).

In recent years, transcriptome analysis of the mycorrhizal plants using microarrays or RNA sequencing has become a most powerful method for detecting changes in gene expression occurring in the host plant as a result of symbiosis. Transcriptional profiling of various AMF-colonized hosts, such as *Medicago truncatula* (Mohammadi-Dehcheshmeh et al. 2018), *Lotus japonicus* (Handa et al. 2015), *Oryza sativa* (Gutjahr et al. 2008; Wang et al. 2020), *Glycine max* (Marquez et al. 2018), *Helianthus annus* (Vangelisti et al. 2018) and *Triticum durum* (Puccio et al. 2023), has enabled the identification of numerous regulatory and functional genes involved in the symbiotic pathways conserved since ancient times. Importantly, the existence of a common symbiosis pathway (CSP) was confirmed and its crucial components were described, which are partly common to AMF and rhizobia (Genre and Russo 2016; Shi et al. 2023). However, the understanding of the CSP and its functioning during mycorrhiza establishment remains incomplete. Alongside well-known contributors of CSP, i.e. leucine-rich repeat receptor kinases, cation channels, nucleoporins, calcium/calmodulin-dependent protein kinase (CCaMK), and CYCLOPS/IPD3 (Genre and Russo 2016), the mycorrhiza-specific factors, such as the GRAS transcription factor RAM1 (Required for Arbuscular Mycorrhization1) and RAM2 (encoding glycerol-3-phosphate acyl transferase), have also been identified, which are not found in other symbiotic pathways (Gobbato et al. 2012). Moreover, the mycorrhizal pathways for nutrient acquisition have been described and transporters expressed mainly or exclusively during AM have been specified (Harrison et al. 2002; Walder et al. 2015; Sawers et al. 2017; Wang et al. 2020; Rui et al. 2023). Generally, transcriptomics revealed the differential regulation of vital genes from various functional categories in mycorrhizal plants. These categories included post-translational regulation, signaling, hormone metabolism, lipid and carbohydrate metabolism, transport and responses to environmental stressors (Zouari et al. 2014; Cervantes-Gámez et al. 2016).

Potato is not typically used as a model plant for mycorrhiza studies, therefore our understanding of the symbiotic pathways in this host is limited. Similarly, there is a little information regarding potato’s defense responses triggered by AMF at the molecular level. Research employing quantitative PCR methods has identified a marker gene in potato root colonization, which plays a crucial role in the symbiotic pathway for nutrient exchange (Gallou et al. 2010). Furthermore, only a few genes associated with the mycorrhizal priming of potato’s defense mechanisms have been described (Gallou et al. 2011). In addition, the transcriptomic data detailing gene expression changes in mycorrhizal potato under the influence of a virus like PVY have not been previously available. Given our prior research demonstrating that AMF can mitigate the detrimental effects of PVY on potatoes (Deja-Sikora et al. 2023), this study aimed to uncover the molecular determinants responsible for the advantages that mycorrhizal associations offer to the plant. To achieve this, we employed a transcriptomic approach and conducted a functional annotation of differentially expressed transcripts to elucidate the components of the symbiotic pathways in mycorrhizal potatoes. Furthermore, we sought to clarify the reasons behind the improved photosynthetic capacity and, notably, the reduced oxidative stress observed in virus-infected potatoes following the establishment of symbiosis. Through analysis of differential gene expression (DGE) profiles, we tried to identify the genes whose overexpression may play a pivotal role in defense strategies within mycorrhizal plants. Lastly, we expected that the results of this study would improve our understanding of the beneficial role of mycorrhiza.

## Materials and methods

### Design of the experiment

The design of the experiment was described in details by Deja-Sikora et al. (2023). Briefly, PVY-infected and PVY-free plantlets of potato (*S. tuberosum* cv. Pirol) utilized in the experiment were obtained from in vitro cultures. The lineage of plants permanently infected by PVY was maintained under sterile conditions for an extended period (approximately 1 year). Once plantlets were acclimated, each plant was transferred to a pot filled with sterile sand to initiate the experiment. The pots were organized into pairs, with one pot containing a PVY-free (PVY-) potato plant and the other pot containing a PVY-infected (PVY +) potato plant. Both plants from each pair were placed within the same isolated tray compartment. Control plants were cultivated without fungi, while the remaining plants were inoculated with spores of two species of AMF (*F. mosseae* and *R. irregularis*). Consequently, six experimental treatments were obtained, including two controls designated as P^PVY−^ and P^PVY+^, and four treatments inoculated with the two AMF species, labeled as P^PVY−^/ Fm, P^PVY+^/ Fm, P^PVY−^/ Ri, and P^PVY+^/ Ri. The experiment lasted 12 weeks. Then, the leaves and roots for qPCR and transcriptomic analysis were collected from each treatment and stored in – 80 °C. Plant growth parameters, oxidative stress indicators (H_2_O_2_, glutathione, ascorbate, lipid peroxidation), and photosynthetic capacity were measured and discussed by Deja-Sikora et al. (2023).

### Extraction of total RNA

RNA for RT-qPCR and transcriptomic analysis was isolated with standard chaotropic salt-phenol–chloroform method. 100 mg of plant tissue (root or leaf) was homogenized in liquid nitrogen. The powder was transferred to 1 ml RNA Extracol (Eurx, Gdansk, Poland), then 0.2 ml of chloroform was added. Aqueous phase was extracted with equal volume of acid Phe/Chl/IAA (25:24:1) and then equal volume of Chl/IAA. RNA was precipitated with 2 volumes of 96% ethanol and washed twice with 1 ml 75% ethanol. Dry RNA pellet was resuspended in 50 µl mixture containing 1 × reaction buffer with MgCl_2_ and 5 U DNase I (Eurx, Gdansk, Poland). The reaction was incubated for 30 min. After DNase treatment RNA purification was done by adding 250 µl 96% ethanol and 250 µl 8 M guanidine HCl (Sigma-Aldrich, Saint Louis, MO, USA) in RNase-free water. The mixture was transferred to the CleanEasy Mini Spin column in collection tube (Canvax Biotech, Kordoba, Spain) and centrifuged for 2 min at 8000 g and RT. The flow-through was discarded and 500 µl of 3 M sodium acetate (pH 5.0) was added to the column and centrifuged. The filter was washed twice with 500 µl of washing buffer (25 mM Tris–Cl pH 7.0 in 75% ethanol). Column was dried by centrifugation for 2 min at 8000 g and RT. RNA was eluted from the column with 50 µl of RNase-free water. The concentration and quality of RNA was checked with NanoDrop 2000 (Thermo Fisher Scientific, Waltham, MA, USA).

### RT-qPCR analysis and mycorrhiza level

Quantitative PCR was used to check the relative number of PVY genome (based on CP gene sequence, see explanation below Table 2) and the relative expression of AMF-induced genes (StPR1, StPR2 and StPT3) in the host plant. Two housekeeping genes of potato (StTUB and StEF1) were used for normalization of expression levels (Fuentes et al. 2016). Primers used in this assay were given in Table 1. For each primer set the efficiency of amplification (always above 1.90, which stands for > 90%) was determined based on 5-point standard curve calculated with a ‘fit points’ method. Reverse transcription of total RNA was performed using Transcriptor First Strand cDNA Synthesis Kit (Roche, Basel, Switzerland). RT reactions were prepared in a final volume of 20 µl containing: 1 × Reverse Transcriptase reaction buffer, 60 µM random hexamer primers, 1 mM dNTPs mix, 20 U of RNase inhibitor, 10 U of Transcriptor High Fidelity Reverse Transcriptase and either 750 ng of leaf RNA or 250 ng of root RNA as a template. The synthesis protocol was as follows: 10 min at 25 °C, 60 min at 50 °C and 5 min at 85 °C. Each reaction was diluted fivefold with nuclease-free water.

**Table 1:** Characteristics of primers used for qPCR analysis including sequences, amplicon lengths and melting temperatures of PCR products

	Primer name and sequence	T_m_ (^o^C)	Amplicon length (bp)	Reference
Reference genes	StTUB.F ATGTTCAGGCGCAAGGCTT	84	101	Nicot et al., 2005; modified
	StTUB.R TCTGCAACCAAATCATTCAT			
	StEF1a.F ATTGGAAACGGATATGCTCCA	84	101	Nicot et al., 2005
	StEF1a.R TCCTTACCTGAACGCCTGTCA			
AMF-induced genes	StPR1.F GGTGCAGGAGAGAACCTT	85	99	Gallou et al., 2010
	StPR1.R GGTACCATAGTTGTAGTTTGGCT			
	StPR2.F TATCATCAGCAGGGTTGCAAA	81	100	Gallou et al., 2010; modified
	StPR2.R TCGCGAAAAATGCCATCTCTAGG			
	StPT3.F GCTTTGCTTACTTATTATTGGCG	82	90	Gallou et al., 2010
	StPT3.R GGAAGCAGCCTTAGTAGCATT			
PVY genome	PVY n2258 GTCGATCACGAAACGCAGACAT	84	179	Lorenzen et al., 2006
	PVY o2439c CCCAAGTTCAGGGCATGCAT			

The qPCR assay was performed using 5 × HOT FIREPol® EvaGreen® qPCR Supermix (Solis BioDyne, Tartu, Estonia). The reactions were prepared in a total volume of 10 µl using 2 µl of diluted RT reaction as a template. The qPCR profile consisted of 40 cycles of 3-step amplification followed by a melting curve step to verify the specificity of amplification. Quantifications were performed in four biological and tree technical replicates for each experimental treatment using Light Cycler 480 (Roche, Basel, Switzerland). Negative reactions were included for each test. The calculation of relative expression was done according to the method by Ng and Ngeow (2022), which gives results consistent with these computed by the REST 2009 software.

The level of mycorrhizal colonization in the potato roots was estimated based on microscopic observation described by Deja-Sikora et al. (2023).

### Transcriptome sequencing

Each sample of DNase-treated total RNA was integrity checked with Agilent RNA 6000 Nano Kit and 2100 Bioanalyzer using Plant RNA Assay (Agilent Technologies, Santa Clara, CA, USA). RNA samples with RIN above 6.5 (for roots) and 8.0 (for leaves) were used for transcriptome sequencing. Transcriptomic libraries were prepared for each sample separately without pooling (8 per one treatment, 48 in total). mRNA enrichment, preparation of double-stranded cDNA libraries, PCR enrichment and sequencing were performed by Novogene Genomic Service (Novogene, Cambridge, UK) using Illumina HiSeq 2500 system (PE150). Raw data were provided as fastq files. Sequencing data were deposited in NCBI BioProject database under the accession numbers: PRJNA809713 and PRJNA809714.

### Bioinformatics analysis

Sequencing reads were corrected with rcorrector v.1.0.4 (Song and Florea 2015) and uncorrectable pairs were removed with FilterUncorrectablePEfastq.py. Adapter trimming was performed with Trim Galore v.0.6.5 (Krueger 2015). Reads coming from ribosomal RNA were filtered out according to by mapping to SILVA v.132 database (SSU and LSU) (Quast et al. 2013) with bowtie2 v.2.4.5 (Langmead and Salzberg 2012). Cleaned reads were mapped to potato genome (DM 1–3 516 R44 v.6.1, obtained from http://spuddb.uga.edu/dm_v6_1_download.shtml) with hisat2 v.2.2.1 (Kim et al. 2019). The resulting.sam files were converted to the bam format with samtools v.1.14 (Danecek et al. 2021) and genome-guided assembly of unannotated transcripts was performed with stringtie v.2.2.0 (Shumate et al. 2022). Assemblies were merged and transcripts quantified with stringtie. The results were formatted for the ballgown R package (Frazee et al. 2015). All computations were carried out on an HP workstation with 16 physical cores (32 threads) and 128 GB RAM. A script enabling computations can be found in Supplementary Material 1 (reads_preprocessing_and_mapping.bash).

Differentially expressed genes (DEGs) were defined as those passing a 0.05 q-value cutoff and having a log2 fold change greater than 1 or smaller than -1 (FC ≥ 2 or FC ≤ 0.5). They were identified with ballgown in R v.4.2.0. Gene enrichment analysis was performed using the topGO package v. 2.50.0 (Alexa and Rahnenfuhrer 2023). Non-metric multidimentional scaling (nMDS) implemented in the metaMDS function of the vegan v.2.6.0 R package was used as an ordination method on Morisita-Horn dissimilarity derived from transcript expression matrix. Significance of grouping was tested with PERMANOVA (Anderson 2017) implemented in vegan’s adonis2 function. All graphs were prepared using standard R graphic functions. A script allowing generation of all figures and tables can be found in Supplementary Material 2 (mycovir_mapped.R).

Protein network analysis was conducted using the STRING database (v11.5) to investigate interactions of the *Solanum tuberosum* protein PAR-1. PAR-1 was identified in the STRING database based on several sequence entries: XP_006358715.1 (NCBI), M1BVE0_SOLTU (UniProt), PF06521 (Pfam), and IPR009489 (InterPro), and then used to explore its protein-protein interactions (PPIs). Interaction networks were validated by examining the PPI enrichment p-value. Identified GO terms, including biological processes, molecular functions, and cellular components, as well as STRING's Local Network Clusters and KEGG pathways, were used to infer the functional roles of interacting proteins. Only interactions with a false discovery rate (FDR) below 0.05 were retained for the final network.

## Results

### The relative expression of StPT3, StPR-1 and StPR-2 genes in mycorrhizal roots of potato

Among the two pathogenesis-related genes investigated in this study, only StPR-1 displayed significant induction across all mycorrhizal treatments (Table 2). *F. mosseae* caused a higher upregulation of StPR-1 compared to *R. irregularis*, with values of 40.75 vs. 21.56 in PVY- and 38.05 vs. 11.73 in PVY + plants, respectively. The presence of the virus was associated with a weaker induction of StPR-1 by *R. irregularis*, with values of 21.56 vs. 11.73. On the other hand, the expression level of StPR-2 remained largely unchanged in response to arbuscular mycorrhiza. However, in the PVY + /Ri treatment, this gene exhibited a potential down-regulation, although the results were inconclusive.

**Table 2:** Relative qPCR analysis of gene expression changes (StPR-1, StPR-2, StPT3) and PVY genome quantification in roots and leaves of mycorrhizal potatoes

	Relative level of gene expression and PVY genomes in roots			
	PVY-negative plants		PVY-positive plants	
	Control vs. Fm	Control vs. Ri	Control vs. Fm	Control vs. Ri
StPR-1	**40.75 / 7.57E-03**	**21.56 / 1.50E-02**	**38.05 / 8.10E-03**	**11.73 / 2.48E-02**
StPR-2	3.80 / 2.55E-01	0.96 / 9.02E-01	1.40 / 3.11E-01	0.41 / 1.65E-01
StPT3	**1124.90 / 1.00E-04**	**2505.99/3.32E-02**	**866.63 / 2.73E-02**	**1291.40 / 9.80E-03**
PVY genome	NA	NA	0.67 / 3.98E-01	0.43 / 3.52E-01
	Relative level of gene expression and PVY genomes in leaves			
StPR-1	2.59 / 4.98E-01	3.05 / 9.29E-02	1.98 / 2.00E-01	1.41 / 5.41E-01
StPR-2	0.75 / 6.07E-01	1.25 / 9.03E-01	1.94 / 9.72E-01	0.17 / 3.14E-01
PVY genome	NA	NA	2.28 / 8.80E-02	3.15 / 5.71E-02
	The level of arbuscular mycorrhiza in roots			
	*F. mosseae*	*R. irregularis*	*F. mosseae*	*R. irregularis*
Mycorrhiza (%)	19.9 ± 3.3 (c)	38.3 ± 3.8 (a)	9.9 ± 8.1 (c)	28.2 ± 1.3 (b)
Arbuscules (%)	1.2 ± 0.1 (b)	11.7 ± 4.5 (a)	0.3 ± 0.2 (c)	1.9 ± 1.6 (bc)

A statistically significant change in expression level within each contrast pair is marked in bold. The fold change is given with the p-value. StPT3 transcripts induced by mycorrhiza were analyzed specifically in roots. PVY genome number, due to its structure – positive-sense ssRNA, perfectly correlates with the number of single-copy CP gene used in qPCR. Letters assigned to the level of arbuscular mycorrhiza indicate statistically significant differences (p ≤ 0.05) between all mycorrhizal treatments, as determined by ANOVA. NA – not analyzed

As expected, the StPT3 gene showed great induction in mycorrhizal plants, with a more pronounced effect observed with *R. irregularis*. Notably, the presence of PVY negatively impacted the mycorrhiza, causing the decrease in StPT3 expression, specifically in the PVY + /Fm treatment from 1124.90 to 866.63, and in the PVY + /Ri treatment from 2505.99 to 1291.40. The levels of arbuscular mycorrhiza and arbuscules based on microscopic observations correlated with folds of StPT3 overexpression found in the qPCR (Table 2).

### The relative abundance of PVY genomes in mycorrhizal roots of potato

In the qPCR assay, it was found that AMF caused a 1.5-fold (*F. mosseae*) to 2.33-fold (*R. irregularis*) reduction in the number of PVY genomes in potato roots (Table 2). These observations coincided with an increase in the number of PVY genomes detected in potato leaves. Specifically, *R. irregularis* was associated with a greater increase compared to *F. mosseae* (3.15 vs 2.28, respectively). However, it’s important to note that these results did not achieve statistical significance, and should therefore be considered as trends rather than definitive findings.

### Summary of transcriptome sequencing output

In total, forty-eight cDNA libraries were prepared using mRNA extracted from both leaves and roots originating from six treatments. These included two control groups (PVY- and PVY +) and four treatment groups, which comprised plants inoculated with either *R. irregularis* or *F. mosseae* (PVY-/Ri, PVY-/Fm, PVY + /Ri, PVY + /Fm). Sequencing generated a total of 598.6 million reads, amounting to approximately 85 gigabytes of data. The number of reads per single library ranged from 9.5 to 18.2 million. High-quality paired reads from each library were aligned to the reference genome of *S. tuberosum* group Phureja DM1-3 v6.1. The percentage of mapped reads in each sample ranged from 41.96 to 90.37%. Lower percentages were specific only for mycorrhizal roots, where fungal and plant transcriptomes were mixed together (Supplementary Table 1). However, we did not confirm that the unmapped reads belonged to the fungal transcriptomes because the reference transcriptomes were unavailable. In control treatments nearly 90% of reads were aligned with the reference. Overall, 180 648 unique transcripts were found in the entire dataset. The result of the transcriptomic analysis were validated by comparing the expression of StPR-1, StPR-2, and StPT3 with the result of qPCR assay (Supplementary Table 2).

### Changes in potato transcriptome upon mycorrhiza and PVY infection

**Fig. 1:**
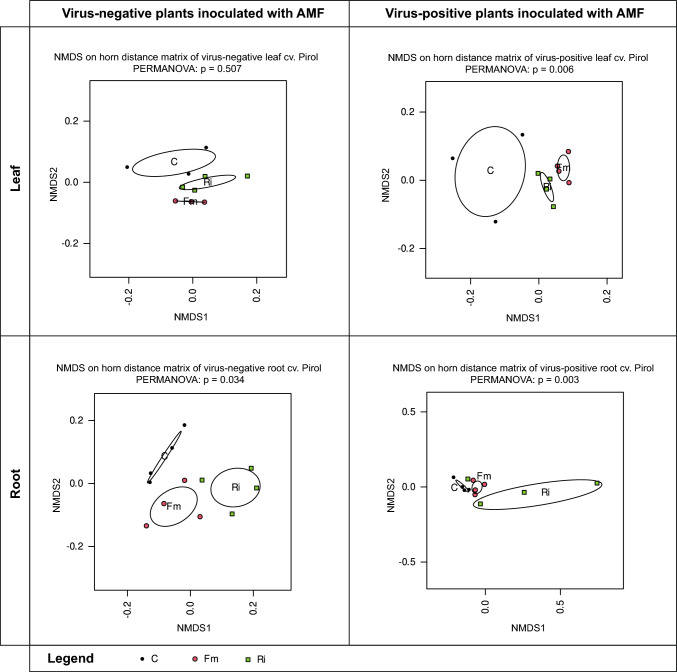
NMDS and PERMANOVA analyses of leaf and root transcriptomes based on Morisita-Horn distance in healthy and PVY-infected potatoes cv Pirol. Ordination plots show the separation of sample groups consisting of control plants non-inoculated with AMF (C, black dots) and mycorrhizal plants colonized by *R. irregularis* (Ri, green squares) or *F. mosseae* (Fm, red dots). Analyzed transcriptomes differed significantly when p < 0.05

mRNA sequencing was employed to investigate gene expression patterns in potatoes under various conditions, including root colonization by two AMF species (*R. irregularis* or *F. mosseae*), PVY infection, or the simultaneous presence of both mycorrhiza and the virus. The resulting transcriptomes were categorized based on the treatment type (PVY-, PVY-/Fm, PVY-/Ri, PVY + , PVY + /Fm, and PVY + /Ri) and the plant organ (leaf or root) from which they originated. An ordination analysis (NMDS) of the expression data using Horn-Morisita dissimilarity revealed significant differences (PERMANOVA p-value ≤ 0.05) between root transcriptomes of control plants and those inoculated with AMF, both in healthy and PVY-infected hosts (Fig. 1). In contrast, leaf transcriptomes of virus-free plants showed no significant differences between control and mycorrhizal plants, despite separate clusters representing each treatment in the ordination space. This outcome may be attributed to the limited number of samples (only 4) analyzed for each treatment. Conversely, in PVY-positive potatoes, the leaf transcriptomes of analyzed treatments (PVY + , PVY + /Fm and PVY + /Ri) were dissimilar to each other and the differences were statistically significant (Fig. 1).

Differentially expressed genes (DEGs) were defined with the criteria of q-value < 0.05 and a log2 fold-change of ≥ 2.0 or ≤ 0.5, which signifies at least a twofold increase or decrease in transcription levels, respectively. The lists of DEGs, along with their functional annotations and Gene Ontology (GO) terms for each pairwise comparison, can be found in Supplementary Table 3. The number of DEGs identified in the transcriptomes of specific experimental treatments was significantly influenced by two key factors: the plant organ under analysis (leaf or root) and the specific AMF species colonizing the plant roots (Table 3). Interestingly, the presence of the virus did not appear to induce significant transcriptional changes in either potato leaves or roots. In fact, the only DEGs identified in the PVY-/PVY + comparison originated from the PVY genome. On the other hand, the plant’s response to mycorrhiza was more prominent and primarily observed in the roots of both, healthy and virus-infected treatments.

**Table 3:** Number of transcripts differentially expressed in leaves and roots of potatoes. The contrast pairs are divided into two groups depending on the virus presence and the mycorrhizal colonization. Statistically significant changes in expression levels included: ≥ twofold change for up-regulation and ≤ 0.5-fold change for down-regulation

Contrast pair	Transcripts in leaves	
	Up-regulated	Down-regulated
Variable: PVY presence		
PVY- /PVY +	3	1
PVY- Fm /PVY + Fm	2	7
PVY- Ri /PVY + Ri	0	0
Variable: AMF presence		
PVY- /PVY- Fm	0	0
PVY- /PVY- Ri	6 (5)	6 (3)
PVY + /PVY + Fm	0	0
PVY + /PVY + Ri	0	0
Contrast pair	Transcripts in roots	
	Up-regulated	Down-regulated
Variable: PVY presence		
PVY- /PVY +	1	4
PVY- Fm /PVY + Fm	7	8
PVY- Ri /PVY + Ri	2	3
Variable: AMF presence		
PVY- /PVY- Fm	121 (72)	25 (20)
PVY- /PVY- Ri	336 (174)	170 (120)
PVY + /PVY + Fm	434 (239)	74 (52)
PVY + /PVY + Ri	203 (104)	71 (53)

The value in parenthesis indicates the number of protein-encoding transcripts identified based on the reference *S. tuberosum* genome

**Fig. 2:**
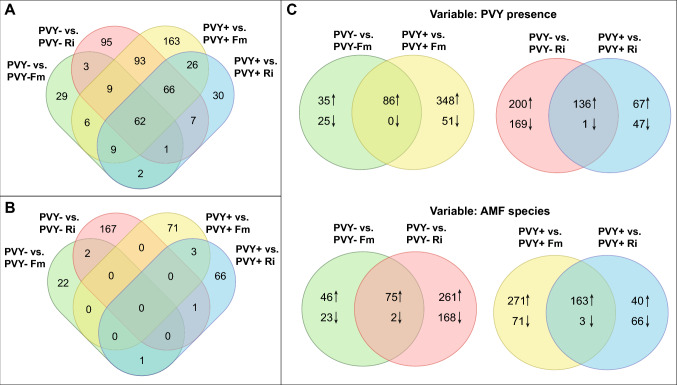
Venn diagrams illustrating the number of differentially expressed genes (DEGs) shared between contrast pairs, comprising the control treatment (PVY- or PVY +) compared with the mycorrhizal treatment (Ri—*R. irregularis* or Fm—*F. mosseae*). The numbers of up-regulated and down-regulated DEGs, indicating similarities and differences across all analyzed pairs, are presented in Panels A and B, respectively. Panel C depicts pairs compared regarding PVY presence or AMF species used for plant inoculation. In Panel C, the top arrow (↑) indicates upregulated DEGs, and the down arrow (↓) indicates downregulated DEGs

Irrespective of the analyzed pairs, mycorrhizal roots consistently exhibited a higher number of up-regulated DEGs compared to down-regulated ones. The greatest up-regulation was observed in PVY + potato plants that were inoculated with *F. mosseae*, with overexpression of 434 DEGs, including 239 protein-coding transcripts. In the same treatment, only 74 transcripts were down-regulated. Healthy plants colonized by *F. mosseae* exhibited up-regulation of 121 DEGs and down-regulation of 25 DEGs. Surprisingly, a different pattern emerged in treatments inoculated with the other AMF species, *R. irregularis*, where PVY- Ri plants had 336 up-regulated DEGs compared to 203 in PVY + Ri plants (Table 3). Venn diagrams highlighted that 62 DEGs were consistently overexpressed in all mycorrhizal plants, both PVY- and PVY + (Fig. 2A). Intriguingly, down-regulated DEGs appeared to be specific to individual treatments, suggesting that mycorrhiza reduced the levels of different transcripts depending on the AMF species and the presence of PVY (Fig. 2B–C).

**Fig. 3:**
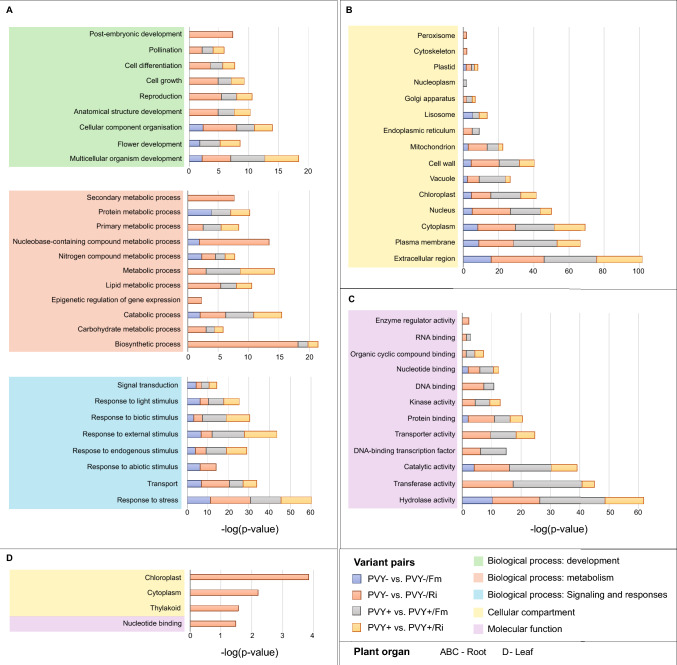
Gene Ontology (GO) term enrichment analysis presenting significantly enriched GOs in root transcriptomes of mycorrhizal palnts (Ri—*R. irregularis* and Fm – *F. mosseae*) compared with control plants (PVY- and PVY +). GO terms were selected based on FDR < 0.01. GOs belonging to different categories are given in the following colors: Biological Processes: development in green (panel A), Biological process: metabolism in red (panel A), Biological process: signaling and responses in blue (panel A), Cellular Components in yellow (panel B) and Molecular function (panel C) in purple. Additionally, panel D shows several GOs enriched in leaf transcriptome of healthy potato inoculated with *R. irregularis*

The GO enrichment analysis revealed that biological processes significantly overrepresented in root transcriptomes across all treatments were primarily associated with stress response (GO:0006950), response to external stimuli (GO:0009605), and transport (GO:0006810) (Fig. 3A). Furthermore, additional categories, including biosynthetic process (GO:0009058), secondary metabolic process (GO:0019748), and post-embryonic development (GO:0009791), were notably frequent in PVY- plants colonized by *R. irregularis*. In terms of subcellular localization, consistent predictions for DEGs across all treatments included extracellular region, cytoplasm, plasma membrane, nucleus, cell wall, and chloroplast (Fig. 3B). The last location was also found to be a main target for DEGs identified in leaf transcriptome of PVY- Ri treatment (Fig. 3D). Regarding molecular function analysis, DEGs were predominantly enriched in activities related to hydrolases, transferases, and transporters (Fig. 3C).

### Analysis of up- and down-regulated root transcripts

**Fig. 4:**
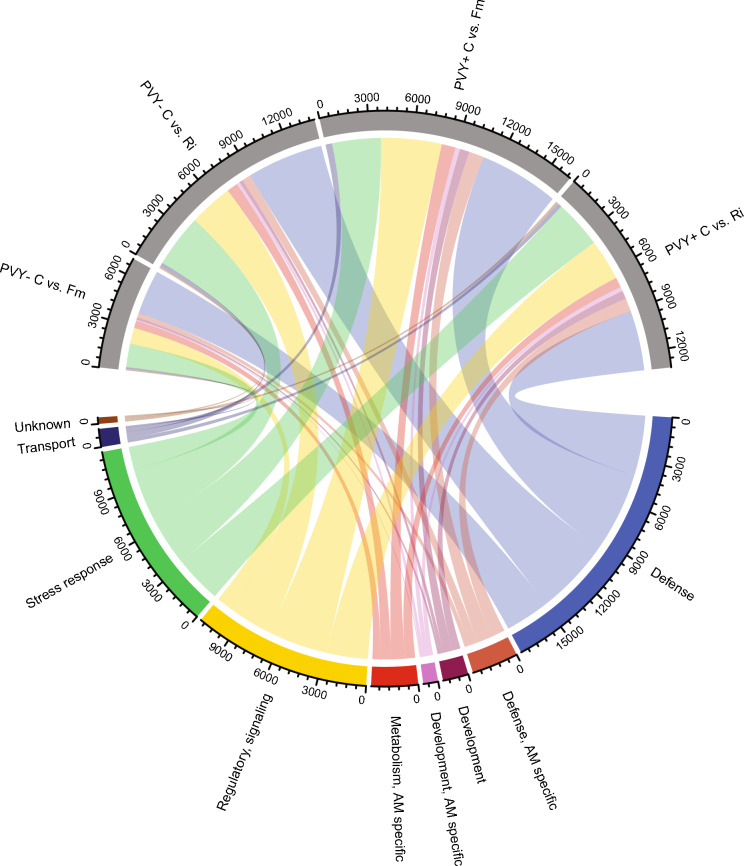
Chord diagram presenting distribution of functions associated with greatly upregulated DEGs across the tretament pairs consisting of non-inoculated control plants compared with these inoculated with AMF (Ri–*R. irregularis* and *Fm–F. mosseae*). DEGs assigned to the specific functions were grouped, then normalized to the summation of the fold-change values, and the relative contribution of each function to a treatment pair (represented by the width for links) was illustrated in the plot

In order to understand the biological significance of the molecular changes occurring in potatoes during interactions with two different species of AMF and PVY, we conducted a functional identification of both up- and down-regulated DEGs within each treatment. While many DEGs were successfully associated with their corresponding proteins, the most significantly overexpressed transcript (MSTRG.67947.1, with a fold change ranging from 1780 to 2882) could not be functionally annotated. The identified transcripts that exhibited substantial up-regulation across all treatments coded for proteins directly involved in stress responses and defense mechanisms (Fig. 4). Notably, among these were cupredoxins/blue-copper proteins, photoassimilate-responsive proteins PAR-1, defensins (PR-12), germins (GLPs, PR-16), non-specific lipid transfer proteins (nsLTPs, similar to PR-14), kiwellins (Barwin-like endoglucanases, PR-4), type III chitinases (PR-8), rhicadhesin receptor-like GLPs, glutathione-S-transferases, Gnk2-homologous domain-containing protein, CASP-like/pathogen-induced membrane protein 1 (PIMP1), disease resistance RPP13-like protein 1, and pathogenesis-related protein PR-10 (Supplementary Fig. 1, Supplementary Table 3). Particularly interesting was the stronger expression of both PR-10 and beta-1,3-glucanase (PR-2) in the presence of mycorrhiza and PVY compared to the PVY + control treatment. Peroxidase, another protein important for plant defense against pathogens, was overexpressed across all mycorrhizal treatments, excluding PVY- Fm.

The other highly enriched DEGs encoded regulatory factors, such as LysM proteins and UDP-glycosyltransferases. Besides these, Sigma factor SigB regulation protein RsbQ, GRAS family transcription factor and serine-threonine protein kinase were also up-regulated in at least three treatments. Myb family transcription factor, C2HC zinc finger protein and syntaxin/t-SNARE protein were overexpressed more specifically, depending on the treatment type.

We observed the overrepresentation of DEGs specifically contributing to arbuscular mycorrhiza development, including GPI ethanolamine phosphate transferase, DUF538, serine carboxypeptidase (SCP1) and subtilase (PR-7). Surprisingly, three of the AM marker proteins involved in lipid metabolism, i.e., beta-keto-acyl ACP synthase I (KASI), fatty acyl-ACP thioesterase (FatM) and RAM2, were up-regulated only in two treatments, PVY- Ri and PVY + Fm. Besides, DEGs encoding subtilisin-like serine endopeptidases required for AM symbiosis development were also found in these treatments. Nevertheless, the overexpression of the high-affinity inorganic phosphate transporter specifically induced by AMF, StPT4, was detected in all mycorrhizal plants. In the case of other Pi transporters, such as StPT3 and StPT5, upregulation was observed in three out of the four analyzed treatments. In most instances, we also confirmed the over 30-fold enrichment of the nitrate transporter NRT1/PTR FAMILY 4.5-like. The up-regulation of other nitrate transporters, including NRT1/PTR FAMILY 1.1-like and NRT1/PTR FAMILY 8.2-like, occurred to a lesser extent. In addition, the PVY + Ri treatment also showed at least 45-fold overexpression of both, the mycorrhizal-specific ammonium transporter and high-affinity nitrate transporter NRT2.4.

Among the enriched transcripts associated with metabolism and developmental changes, we identified genes coding for cytochrome P450 and cytochrome b561. The latter appeared to be specifically induced by *F. mosseae*. Additionally, increased levels of gamma vacuolar processing enzymes, ripening-related protein 1 (RRP1), senescence-specific cysteine protease SAG39-like, and gibberellin 3-beta-dioxygenase were detected in at least three of the mycorrhizal hosts. Gibberellin 20 oxidase was upregulated only in PVY + Fm. Regarding factors that may contribute to enhanced photosynthesis efficiency, we observed the overexpression of pentatricopeptide repeat protein (PRP) and 15-cis-phytoene desaturase (the latter one specifically in PVY + Ri). Notably, some expression changes were also observed in the leaves of mycorrhizal potatoes, particularly in the treatment colonized by *R. irregularis*. Among the enriched transcripts in this context were those encoding Photosystem I reaction center subunit XI (fold change 3637), a key component of the chloroplast thylakoid membrane, and FMN-linked oxidoreductase (fold change 7.8) (Supplementary Table 3).

Down-regulated DEGs displayed specificity for each treatment, with only a few overlapping occurrences. Significantly reduced expression of 2-oxoglutarate (2OG) and Fe(II)-dependent oxygenase was observed in three treatments, excluding PVY- Ri. Additionally, the transcript for papain-like cysteine protease was notably underrepresented in both PVY- mycorrhizal roots, irrespective of the AMF species colonizing the plant. On the other hand, the expression of the chloroplastic STAY-GREEN 1 protein was down-regulated in both PVY + treatments. Interestingly, several DEGs encoding peroxidases exhibited underexpression in PVY- Ri roots. In the same treatment, several DEGs associated with regulatory functions were expressed at significantly lower levels, including histones H1F, H2B, H3.3, and H4. Similarly, down-regulation was observed in certain ribosomal proteins and RNA-binding proteins. The full list of down-regulated DEGs with functional annotations is given in Supplementary Table 3.

### Network analysis for selected overexpressed proteins

**Fig. 5:**
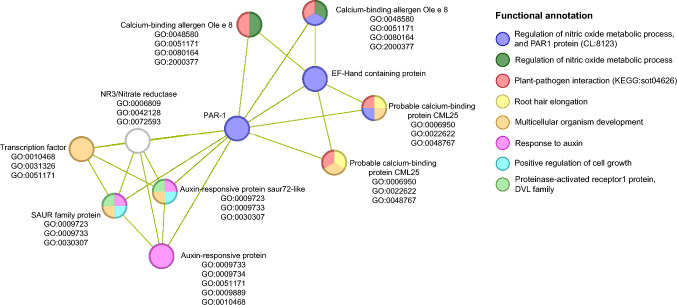
Network analysis for photoassimilate-responsive protein (PAR-1) greatly upregulated in mycorrhizal potato cv Pirol. The hypothetic network including proteins that may possibly interact with PAR-1 is based on in silico analysis using STRING database v11.5 (PPI enrichment p-value 0.00123). Interacting proteins were assigned GO terms for Biological Processes: GO:0048580 Regulation of post-embryonic development; GO:0051171 Regulation of nitrogen compound metabolic process; GO:0080164 Regulation of nitric oxide metabolic process; GO:2,000,377 Regulation of reactive oxygen species metabolic process; GO:0006950 Response to stress; GO:0022622 Root system development; GO:0048767 Root hair elongation; GO:0009723 Response to ethylene; GO:0009733 Response to auxin; GO:0030307 Positive regulation of cell growth; GO:0009734 Auxin-activated signaling pathway; GO:0009889 Regulation of biosynthetic process; GO:0010468Regulation of gene expression; GO:0010468Regulation of gene expression; GO:0031326 Regulation of cellular biosynthetic process; GO:0006809 Nitric oxide biosynthetic process; GO:0042128 Nitrate assimilation; GO:0072593 Reactive oxygen species metabolic process. Colors in the figure represent functional annotations based on the integration of GO terms, STRING clusters (CL), and KEGG pathways

Among several protein-encoding DEGs exhibiting significant up-regulation in mycorrhizal potato plants, photoassimilate-responsive proteins PAR-1 (fold change: 634–2642) are of particular interest as they are poorly characterized, both in terms of function and participation in cellular processes. Using the STRING database v11.5 we performed network analysis to unveil the links between PAR-1 (PGSC0003DMT400053728) and various processes, such as stress and defense responses. The resulting network (PPI enrichment p-value 0.00123) suggested that PAR-1 due to possible interaction with the calcium-binding allergen Ole e 8 may be involved in the regulation of nitric oxide metabolism and plant-pathogen responses (Fig. 5). Additionally, the interaction of PAR-1 with CML25 appears to be associated with both plant-pathogen responses and root hair elongation. Interestingly, the possible interaction of PAR-1 with several auxin-responsive proteins may contribute to the positive regulation of plant growth.

## Discussion

The results presented in this study build upon the experiments described by Deja-Sikora et al. (2023). In our previous work, we characterized the visible symptoms of PVY infection in the potato cultivar Pirol, which included a mosaic pattern, crinkling, and stunting of leaves, but no necrotic spots or tuber disease (Supplementary Fig. 2). By studying the impact of the virus on plant growth parameters, we confirmed that the pathogen was fully functional in the host. Furthermore, we found that the establishment of arbuscular mycorrhiza in the roots of PVY-infected potatoes significantly increased tuber yields. AMF mitigated the oxidative stress induced by the virus and improved the photosynthetic capacity of plants affected by PVY. Interestingly, microscopic examinations of stained fungal structures revealed that the virus had a negative impact on AMF, reducing the level of root colonization and the frequency of arbuscules. Additionally, ELISA assays indicated that arbuscular mycorrhiza decreased PVY content in plant roots while simultaneously favoring virus accumulation in plant leaves. These findings suggested the existence of a specific interaction between AMF and PVY. To gain a more comprehensive understanding of these observations, we conducted more advanced analyses, including qPCR and transcriptome sequencing, which enabled the examination of molecular factors involved in the mycorrhiza-virus interplay.

### Persistent PVY infection in potato can result in stagnant host–pathogen interaction

The comparative analysis of PVY- vs. PVY + transcriptomes in both potato roots and leaves showed no significant changes in host gene expression patterns upon virus presence. In fact, the only DEGs detected in this contrast pair were the PVY genomes (positive-sense ssRNA), confirming systemic infection in the host. This phenomenon, although not thoroughly understood, was previously described by Honjo et al. (2020), who highlighted seasonality in viral dynamics and virus–host interactions in the model consisting of *Arabidopsis halleri* permanently infected by Turnip mosaic virus. The authors found that long-term host–pathogen interactions through vertical transmission from maternal plant to the clonal offspring could promote low virus virulence and high host resistance. Persistent infection may result in low virus loads on host plants as long as a single virus lineage exploits its own host for longer periods. Furthermore, Honjo et al. (2020) noticed that in summer season due to temperature stabilization, plant-virus interaction may enter a stagnant phase, during which no DEGs can be detected despite high virus accumulation. The authors attributed this finding to the existence of abiotic/biotic determinants of gene expression other than the virus. We suggest that that potato-PVY interaction in our experimental model, due to persistent viral infection and incubation at a temperature of 18 °C, could have entered a similar phase, resulting in the detection of PVY accumulation only. However, the temperature applied during this experiment was chosen to be optimal for potato growth and establishment of arbuscular mycorrhiza, which most effectively develops at 20–26 °C (Kilpeläinen et al. 2020).

### AMF species induce symbiosis-related genes to various degree in the presence of PVY

In our investigation, we focused on the expression of AM-specific genes, namely phosphate transporter 3 (PT3) and two pathogenesis-related proteins (PR-1 and PR-2), which are known to play roles in various stages of symbiosis establishment (Gallou et al. 2010). PT3, as identified by Rausch et al. (2001), is localized specifically in arbuscule-containing root cells of potato. Since PT3 has been confirmed as a marker for functional AM in *S. tuberosum*, *S. lycopersicum*, and *L. japonicus* (Maeda et al. 2006; Gallou et al. 2011; Miozzi et al. 2011; Ruzicka et al. 2012), we employed the expression of the StPT3 gene to molecularly evaluate the level of arbuscules in healthy and PVY-infected host plants colonized by either *F. mosseae* or *R. irregularis*. As expected, StPT3 was significantly upregulated across mycorrhizal treatments, and the levels of gene expression corresponded positively with the frequencies of arbuscules found in microscopic analysis (Deja-Sikora et al. 2023). *R. irregularis* could colonize potato roots more robustly than *F. mosseae*, as reflected in the higher content of arbuscules and the greater induction of the StPT3 gene. However, the level of StPT3 transcripts decreased in the presence of PVY. The virus consistently appeared to reduce the activity of the AMF-plant interface for nutrient exchange in potato roots. There are limited studies regarding the impact of phytoviruses on AM, but a similar phenomenon was reported by Khoshkhatti et al. (2020), who showed that in cases where viral infection preceded mycorrhiza formation, the level of fungal colonization was reduced. Nevertheless, the molecular determinants behind this observation remain unknown. It is possible that the overexpression of glucan endo-1,3-beta-glucosidase VI (other than PR-2), found only in virus-infected mycorrhizal plants, may contribute to the regulation of symbiosis levels. This finding is supported by results published by Lambais and Mehdy (1998), who demonstrated that beta-1,3-glucanase mRNA accumulated around numerous cortical cells containing arbuscules, specifically in conditions of low P concentration. They suggested that the localized accumulations of endochitinase and beta-1,3-glucanase mRNAs might be involved in controlling intraradical fungal growth by facilitating or restricting the colonization.

### AMF species differently influence the accumulation of PVY in potato leaves and roots

Transcriptomic analysis, along with qPCR, provided further confirmation of our previous findings regarding the influence of AMF on PVY levels in both roots and leaves. In line with our observations, many other studies have reported an increase in virus content in the leaves of mycorrhizal plants. For instance, tomato mosaic virus (TMV)-infected tomatoes associated with *Rhizoglomus irregulare* or tomato spotted wilt virus (TSWV)-infected tomatoes colonized by *Glomus mosseae* have exhibited higher virus content in leaves (Miozzi et al. 2011; Khoshkhatti et al. 2020). However, the concentration of the virus in mycorrhizal roots had not been previously investigated. In the research by Deja-Sikora et al. (2023), we were the first to highlight the reduced presence of PVY in potato roots when interacting with *R. regularis* or *F. mosseae*. The specific mechanism inhibiting virus multiplication in mycorrhizal roots remains unclear. Nevertheless, our transcriptomic analysis showed a significant increase in the expression of a pathogenesis-related protein belonging to the Bet v 1 (PR-10) protein family, as well as flavonoid O-methyltransferase in the roots of PVY-infected plants compared to healthy plants. PR-10 proteins are known to play a role in regulating the biosynthesis of flavonoids and have the capacity to bind various compounds including flavonoids, sterols, and cytokinins with high affinity (Casañal et al. 2013; Morris et al. 2021). Notably, higher concentrations of flavonoids in root exudates have been shown to enhance the symbiotic relationship between AMF and host plants (Tian et al. 2021). Moreover, increased accumulation of flavonoids has been confirmed in the mycorrhizal roots of *M. truncatula* at various stages of symbiosis (Schliemann et al. 2008). On the other hand, flavonoids have been documented to possess antiviral properties by hampering virus genome replication or protein translation (Chojnacka et al. 2021; Anikina et al. 2023). Hence, it’s possible that the increased presence of these compounds in mycorrhizal roots may interfere with virus activity. Consequently, reduced load of PVY in potato roots may be one of the factors underlaying the increased tuber yield in mycorrhizal PVY + plants. As demonstrated in our previous work (Deja-Sikora et al. 2023), the virus itself dramatically reduced tuber development, while AMF alleviated the effect of the pathogen. The extent of mitigation varied among fungal species, with *R. irregularis* proving the most effective. This species also achieved a higher level of mycorrhization in PVY + roots, further confirming that plant-AMF compatibility may play a crucial role in modulating the observed results.

Interestingly, several mechanisms linking specific virus accumulation in plant leaves with arbuscular mycorrhiza have been proposed (Hao et al. 2019). One hypothesis suggested that viral replication is stimulated due to the increased availability of phosphorus in plants expressing mycorrhiza-inducible inorganic phosphate transporter PT4 (Daft and Okusanya 1973; Lacroix et al. 2017). Another mechanism might involve the downregulation of specific defense factors, such as PR proteins, heat-shock (HS) proteins, and glutathione S-transferase (GST) in the presence of a virus (Miozzi et al. 2011). In our study, we indeed observed a significant upregulation of several Pi transporters, including PT3, PT4 and PT5, but this occurred only in the transcriptomes of potato roots. Among these transporters, PT5 was more specifically overexpressed in the PVY + plants. We did not find any changes in the expression of Pi transporters in the leaves. Nevertheless, it appeared that potatoes interacting with AMF had an improved supply of Pi. Furthermore, we did not observe a decreased expression of the defense factors mentioned above in the transcriptomes of virus-infected plants, neither in the roots nor in the leaves. Since we did not find any other evidence to clearly link molecular determinants with virus multiplication in leaves, it is possible that the nutritional status of the plants played a dominant role in this phenomenon.

### Arbuscular mycorrhiza induces defense-related genes in potato

We noticed the overexpression of several defense proteins in mycorrhizal potatoes cv Pirol, both in healthy and PVY-infected plants. Among these were intra- and extracellular pathogenesis-related proteins (PRs), which are key contributors to the plant’s immune response, such as systemic acquired resistance (SAR), activated during interactions with various pathogens (Ali et al. 2018; Goddard et al. 2021; Lopes et al. 2023). According to current knowledge, PRs are grouped into 17 families (PR-1 to PR-17) with partially characterized functions. Some of PR proteins act as chitinases, glucanases, peroxidases, defensins, endoproteinases, nucleases, lipid transfer proteins (LTPs), and more (Sels et al. 2008; Lopes et al. 2023). In our study, qPCR revealed the upregulation of PR-1 in all mycorrhizal treatments, even those not affected by PVY. We chose to examine the level of PR-1 transcripts because this gene has previously been shown to be significantly induced by viruses, playing the role in resisting pathogen attacks. Elevated levels of PR-1 have been identified in tobacco and potato infected by PVY, as well as in tobacco challenged by TMV (Naderi and Berger 1997; Akbudak et al. 2020; Amin et al. 2023). Consistent with our findings, several studies have confirmed PR-1 overexpression in mycorrhizal plants when facing pathogens (Gallou et al. 2011; Song et al. 2015; Marquez et al. 2018; Khoshkhatti et al. 2020). This phenomenon is considered an important factor in AMF-primed plant resistance to biotic stressors, supporting the protective role of arbuscular mycorrhiza (Weng et al. 2022). Interestingly, Khoshkhatti et al. (2020) observed that PR-1 expression induced by viral infection in non-mycorrhizal plants decreased over time, while in AMF-associated plants, it remained consistently high. This observation aligns with our findings, as the relative qPCR analysis for PVY- vs. PVY + pair did not reveal significant induction of PR-1 in both non-mycorrhizal treatments. In our study, only plants colonized by *F. mosseae* or *R. irregularis* displayed upregulation of PR-1.

According to our qPCR analysis, the expression of one of the PR-2 genes encoding beta-1,3-endoglucanases was not significantly affected by AMF. Nevertheless, we did find an enrichment of another beta-1,3-endoglucanase exclusively in the transcriptomes of PVY + mycorrhizal plants. The reasons behind this observation remain unclear, as this enzyme plays a role in various processes, including remodeling plant cell walls, mobilizing callose, and breaking down both self and non-self glucan structures. As such, beta-glucanases serve as pivotal regulators in both symbiotic and antagonistic plant–microbe interactions (Perrot et al. 2022). More recently, Chandrasekar et al. (2022) discovered that plant beta-1,3-glucanases are involved in releasing soluble beta-glucans with antioxidant activities from fungal walls. This process is crucial for the roots to successfully establish interactions with beneficial endophytic fungi. Therefore, it’s possible that the induction of beta-1,3-glucanase observed in PVY + /Fm and PVY + /Ri treatments may be linked to the regulation of mycorrhiza levels in these plants.

Plethora of other PR and PR-like genes contributing to plant immune responses were induced at various levels in potatoes cv Pirol interacting with *F. mosseae* or *R. irregularis*. Most greatly overexpressed PRs included defensins (PR-12), major allergens Pru Bet v 1 family (PR-10), non-specific lipid transfer protein (PR-14) and germins (PR-16). Defensins belong to antimicrobial peptides acting against viruses, bacteria and fungi (Sher Khan et al. 2019), but they were not confirmed to negatively impact the root colonization by AMF (de Coninck et al. 2013; Kaur et al. 2017). Interestingly, defensins, to which AMF were insensitive, displayed an antifungal activity against leaf rust pathogen *Puccinia triticina* invading wheat (Kaur et al. 2017). Furthermore, Uhe et al. (2018) showed that mycorrhiza-induced defensins can be involved in late stage of arbuscule functioning and its transition into a post-symbiotic state in *M. truncatula* colonized by *R. irregularis*. It seems that these proteins specialize not only in conferring resistance to pathogenic fungi, but also regulate the development of mycorrhiza. Similarly to defensins, PR-10 appears to be another versatile stress response factor participating in defense pathways activated in response to diverse pathogens, including viruses (Lopes et al. 2023). In addition to the role of PR-10 in the regulation of flavonoid biosynthesis, which was discussed above, Kundu et al. (2019) proposed that PR-10 may participate in salicylic acid (SA) signaling-induced SAR (systemic acquired resistance) during viral infection. In our study, the upregulation of PR-10 was noticed in all mycorrhizal treatments, but in the presence of PVY the overexpression reached twice as much level as in healthy plants, suggesting the important role of this protein in plant-virus interaction. On the other hand, the relationship between the expression of major allergen PR-10 and AMF is not fully characterized. Devers et al. (2011) pointed that the degradation of PR-10 transcripts was an important step during arbuscular mycorrhiza establishment. Nevertheless, we could not find any clear evidence for negative feedback between PR-10 upregulation and mycorrhiza level.

Our comparative analysis of control vs. mycorrhizal plant transcriptomes reveled the significant enrichment of DEGs related to the fatty acid metabolism. We found that along the upregulation of non-specific lipid transfer protein (nsLTP) another proteins, such as HXXXD-type acyltransferase, glycerol-3-phosphate acyltransferase (RAM2) or beta-keto-acyl ACP synthase I (KASI), were overexpressed in mycorrhizal potatoes. nsLTPs, categorized as PR-14, were suggested to be involved in the regulation of the hydrophobic barrier formation as they can contribute to the cutin monomers secretion and deposition (Liu et al. 2015; Wang et al. 2021). The accumulation of cuticular wax, composed of long-chain fatty acids, is one of the mechanisms preventing pathogen infection (Gao et al. 2022). Thus, the role of PR-14 in pathogen defense is stressed. On the other hand, Fonseca-García et al. (2021) discussed the role of nsLTPs in the legume-rhizobium symbiosis, during the early and late stages of nodulation. In legumes, nsLTPs contributed to regulating symbiont entry and promoting root cortex infection. Similarly, Gasser et al. (2022; 2023) reported the upregulation of gene encoding nsLTPs in the symbiotic nodules of *Alnus glutinosa* interacting with *Frankia alni*. Furthermore, based on their lipid transfer function, Gao et al. (2022) speculated that LTPs can play an important role in root colonization by AMF through transferring lipids from host plant to AMF. This conclusion, although not fully confirmed, aligns with the results of our study. We found consistently high upregulation of nsLTP in both, heathy and virus-infected potatoes colonized by AMF, which suggest that this protein may be involved in maintaining symbiosis.

Plant germin-like proteins (PR-16), members of the RmlC-like cupins superfamily, represent another set of mycorrhiza-inducible defense factors well-documented for their antimicrobial properties (Liao et al. 2021). In our study, numerous transcripts encoding GLPs exhibited significant enrichment during the interaction between potatoes and AMF. Handa et al. (2015), in their transcriptional profiling of *L. japonicus* associated with *R. irregularis*, identified LjGLP among the genes markedly induced during mycorrhiza development. Similarly, in a study of *M. truncatula*-*G. intraradices* symbiotic association, Doll et al. (2003) found the upregulation of GLP genes to be a highly conserved mycorrhiza-specific trait. A wealth of evidence in the literature supports the integral role of these proteins in the establishment and maintenance of mycorrhizal symbiotic associations (Hogekamp and Küster 2013; Fiorilli et al. 2018; Ilyas et al. 2022). Notably, several GLPs have been overexpressed in root cells containing arbuscules (Doll et al. 2003; Hogekamp and Küster 2013). Handa et al. (2015) suggested that secretory GLPs, known for their involvement in nonspecific disease resistance, may control fungal infection within the apoplastic space. By supporting mycorrhizal associations, GLPs can indirectly enhance the plant’s resilience to a range of environmental challenges.

It’s well-established that in plant-virus interactions, the activation of host defense mechanisms is associated with an increased production of reactive oxygen species (ROS) (Hernández et al. 2016). In our prior research, we demonstrated that AMF alleviated oxidative damage in PVY-infected potatoes (Deja-Sikora et al. 2023). The transcriptomic analysis now confirms that mycorrhiza indeed enhances the expression of various genes encoding antioxidant proteins. We observed an upregulation of DEGs encoding peroxidase (PR-9) and multiple glutathione-S-transferases (GSTs). Additionally, GLPs (PR-16), highly overexpressed in mycorrhizal potatoes, are known to possess also superoxide dismutase (SOD) activity against superoxide radicals (Liao et al. 2021; Shahwar et al. 2023). Interestingly, transcripts encoding specific GLP isoforms were more enriched in PVY + plants than in PVY-, indicating an increased demand for protection in the presence of the virus. These results are in line with findings from various other studies (Doll et al. 2003; Hogekamp and Küster 2013; Vangelisti et al. 2018), suggesting the mycorrhiza-specific induction of oxidative stress response proteins. On the other hand, Miozzi et al. (2011) observed no activation of GST in TSWV-infected mycorrhizal tomatoes. As information on transcriptomic analyses in plants interacting with both AMF and viruses remains limited, further studies are needed to uncover the factors responsible for these inconsistent observations.

### Overexpression *of PAR*-1 in mycorrhizal potato may constitute another defense factor

The significantly increased level of transcripts encoding PAR-1 in all mycorrhizal plants, regardless of virus infection, is an intriguing phenomenon. Drawing on our previous observations (Deja-Sikora et al. 2023), it seems likely that this phenomenon is a consequence of the enhanced photosynthetic capacity in potatoes colonized by AMF. PAR-1 is poorly characterized, but it is known to play a role in regulating the allocation of photoassimilates, the products of photosynthesis (Herbers et al. 1995). The improved photosynthesis, one of the mycorrhizal benefits, is surely linked to the overexpression of factors responsible for sensing the level of sugar (sucrose or glucose) accumulated in the plant’s leaves. AMF-induced upregulation of PAR-1 has not been reported yet. However, it’s important to consider that PAR-1 can also be a part of the plant’s defense mechanism against virus infection, as suggested by Herbers et al. (1995). Their research on gene expression in PVY-infected tobacco indicated that the overexpression of PAR-1, along with PR genes, could participate in the plant’s immune response to the virus. PAR-1, which exhibits similarities to other PR proteins, might be involved in signaling or metabolic pathways that contribute to the pathogen resistance. Furthermore, Herbers et al. (1995) put forth a hypothesis that the induction of PAR-1 and PR proteins by pathogens might involve similar mechanisms. They noted that viruses can lead to significant disruptions in carbohydrate metabolism in leaves, resulting in the accumulation of starch and soluble sugars. This, in turn, could trigger the overexpression of PAR-1 and PR proteins. While this response seems to assist the plant in resisting PVY, it’s important to recognize that the specific mechanisms of how PAR-1 interacts with PR and the virus are still unclear. Interestingly, our network analysis for PAR-1 suggested a possible link between this protein and the calcium-binding allergen Ole e 8, which is involved in plant-pathogen interactions in *S. lycopersicum* (KEGG pathway sly04626). This analysis indicated that calcium-binding proteins (CaBP) may contribute to the modulation of nitric oxide (NO) metabolism in plants, as also suggested by Lee et al. (2019). Furthermore, there is substantial evidence that NO plays a role in plant defense against viruses, independently of the SA pathway. NO can induce the expression of resistance-related genes, specifically PR-1, which has been observed during long-term host infections by potato virus X (PVX) or TMV (Li et al 2014). NO has been documented to participate in chitosan oligosaccharide-induced resistance to TMV by regulating beta-1,3-glucanase expression (Zhang et al. 2019). In our study, we observed the mycorrhiza-induced overexpression of PAR-1, PR-1, and beta-1,3-glucanase (exclusively in PVY + plants). This finding suggested that NO may be a factor involved in the regulation of gene expression during mycorrhizal interactions, thus enhancing potato resistance to PVY. This is possible because arbuscular mycorrhiza has been found to influence NO metabolism in plants, particularly under stress conditions, such as pathogen attack, which can trigger a hypersensitivity (HR) response in the host (Shah et al. 2023). Our speculation based on in silico analysis requires further experimental confirmation.

### Arbuscular mycorrhiza can influence photosynthetic capacity in PVY-infected potato

Finally, the improved photosynthetic capacity observed in PVY + potatoes colonized by *F. mosseae* or *R. irregularis*, as described by Deja-Sikora et al. (2023), prompted us to investigate the upregulated DEGs for genes that might enhance photosynthesis. However, the leaf transcriptomes displayed substantial similarities, making it difficult to discern significant alterations in gene expression. Despite this, we identified a noteworthy change in the leaf transcriptome of healthy potatoes colonized by *R. irregularis*: a remarkable 3637-fold increase in the expression of a gene encoding the chloroplastic photosystem I reaction center subunit XI. This finding, together with possible GLP-dependent regulation of photosynthetic levels, highlights the ability of mycorrhiza to modulate photosynthesis, providing further insight into our previous observations.

## Conclusions

The results of transcriptomic analysis clarified our previous observations regarding the possible interactions occurring in the tripartite biosystem consisting of potato with permanent PVY infection and AMF. The indirect interaction between AMF and the virus may involve the action of some specific pathogenesis-related proteins, such as PR-10, which are induced in plant roots by the symbiosis. Additionally, a wealth of plant defense genes can be positively regulated by the mycorrhiza. Most of them encode proteins from different PR families including chitinases, endoglucanases, defensins, germins and more. Furthermore, an increased expression of enzymes contributing to oxidative stress mitigation, such as peroxidase and glutathione-S-transferase, appears to be one of key mechanisms activated during plant response to pathogen. Unexpectedly, PAR-1, which was previously proposed to be included to PRs responding to virus infection, was also induced by AMF in the absence of viral pathogen. This finding suggests that PAR-1 may be another defense protein mobilized by mycorrhiza as a part of plant defense pathways, and that both the sugar level and NO may be an important signal factors during plant-pathogen interaction. However, this phenomenon requires further research.

## Supplementary Information

Below is the link to the electronic supplementary material.

Supplementary file1 (XLSX 11 kb)

Supplementary file2 (PDF 1399 kb)

Supplementary file3 (PDF 322 kb)

Supplementary file4 (DOCX 13 kb)

Supplementary file5 (XLSX 53 kb)

Supplementary file6 (BASH 5 kb)

Supplementary file7 (R 29 kb)

## Data Availability

The datasets generated during the current study were deposited in NCBI BioProject database under the accession numbers: PRJNA809713 and PRJNA809714.
