# Supplementary material for: Transcriptomic responses of Solanum tuberosum cv. Pirol to arbuscular mycorrhiza and potato virus Y (PVY) infection
Source: Plant Mol Biol. 2024 Nov 11;114(6):123. doi: 10.1007/s11103-024-01519-9 (PMC11554710; doi:10.1007/s11103-024-01519-9)
Supplement: Supplementary file 2 — Supplementary file2 (PDF 1399 kb) [file 11103_2024_1519_MOESM2_ESM.pdf]

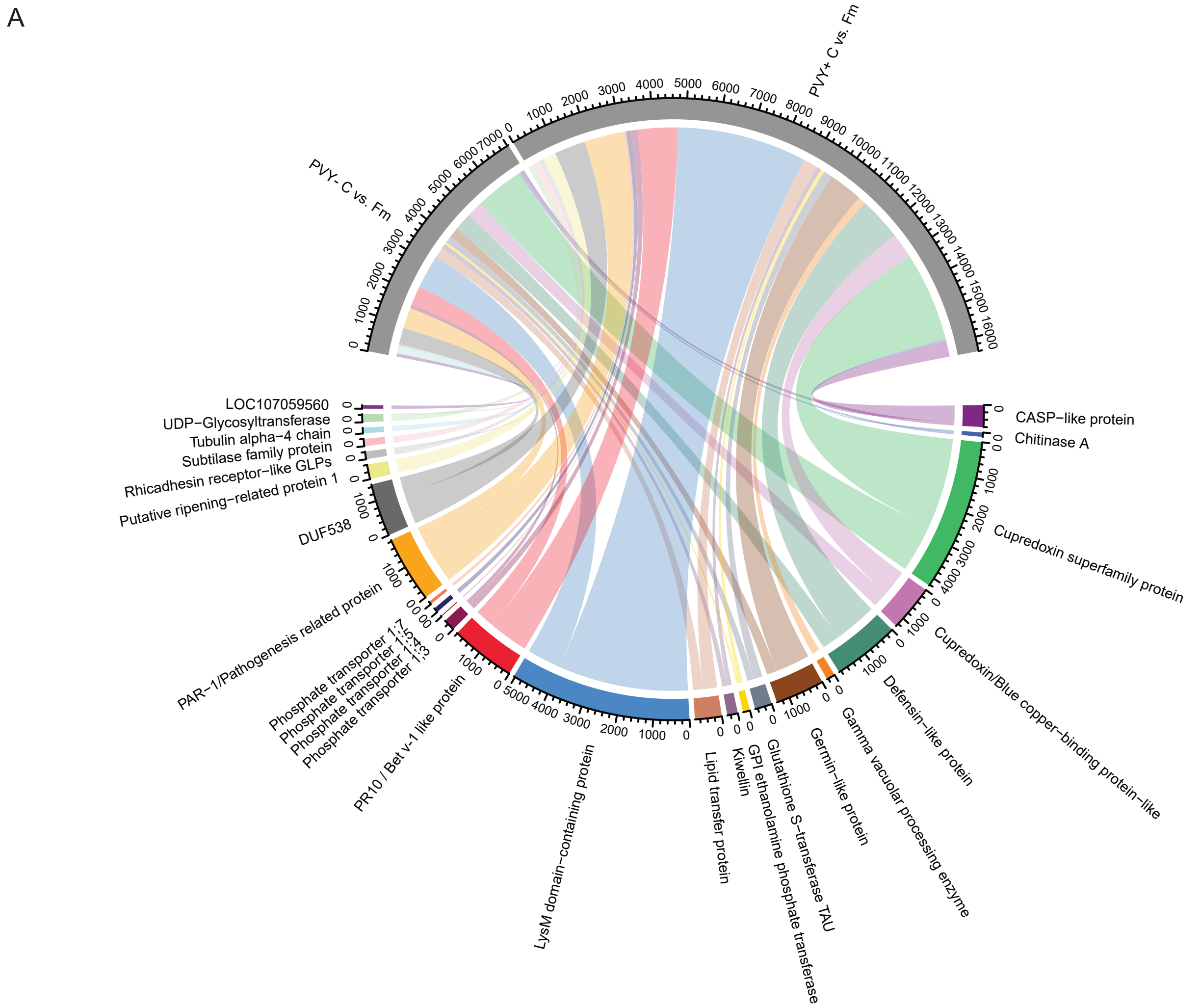

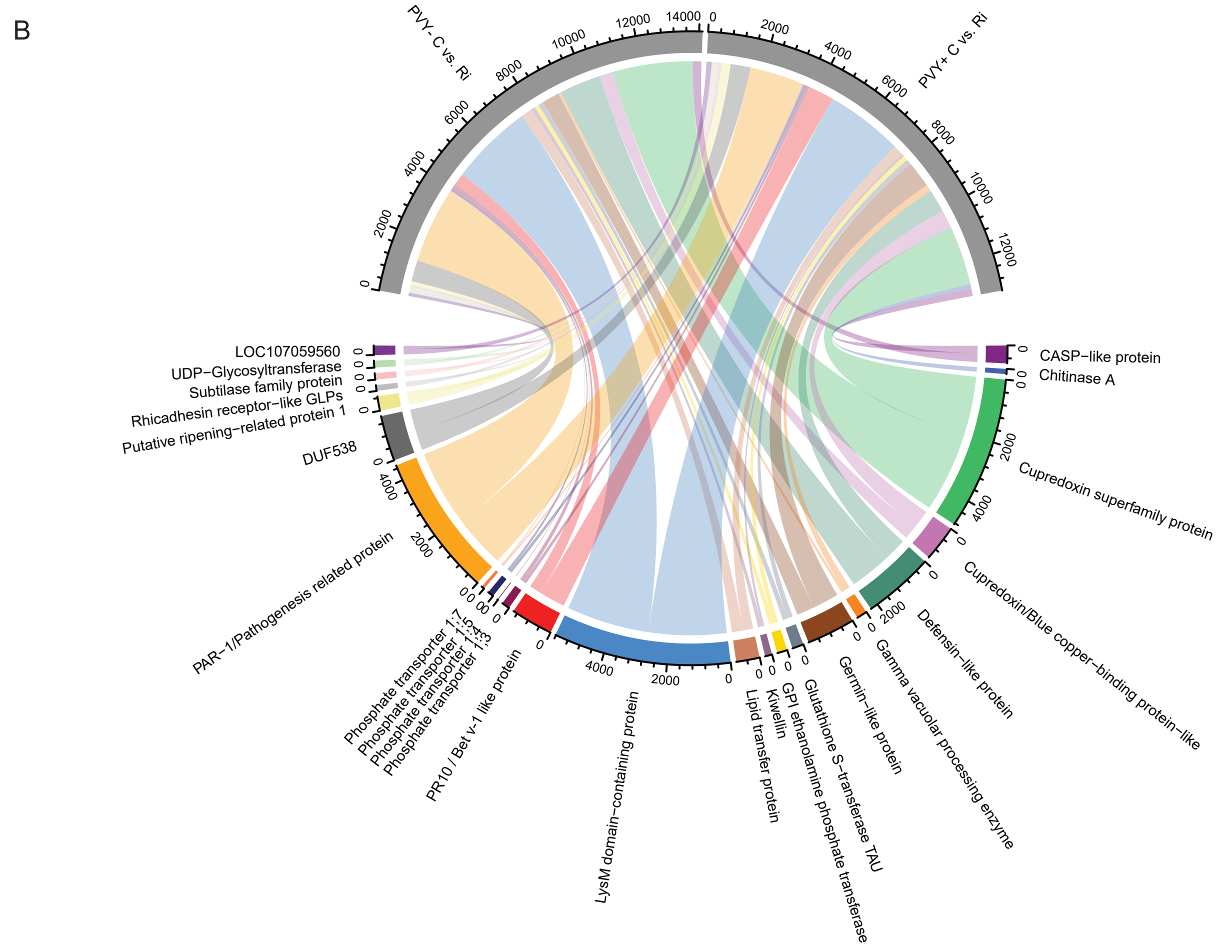

Supplementary figure 1. Chord diagram showing the distribution of most overexpressed DEGs in root transcriptomes across the mycorrhizal variant pairs differing in the presence of PVY. The width of each line is determined by the normalized number of transcript counts. Both AMF species specifically induce similar DEGs irrespective of the viral infection. A - potatoes inoculated with *F. mosseae*; B - potatoes inoculated with *R. irregularis*.
