# Supplementary material for: Transcriptomic responses of Solanum tuberosum cv. Pirol to arbuscular mycorrhiza and potato virus Y (PVY) infection
Source: Plant Mol Biol. 2024 Nov 11;114(6):123. doi: 10.1007/s11103-024-01519-9 (PMC11554710; doi:10.1007/s11103-024-01519-9)
Supplement: Supplementary file 3 — Supplementary file3 (PDF 322 kb) [file 11103_2024_1519_MOESM3_ESM.pdf]

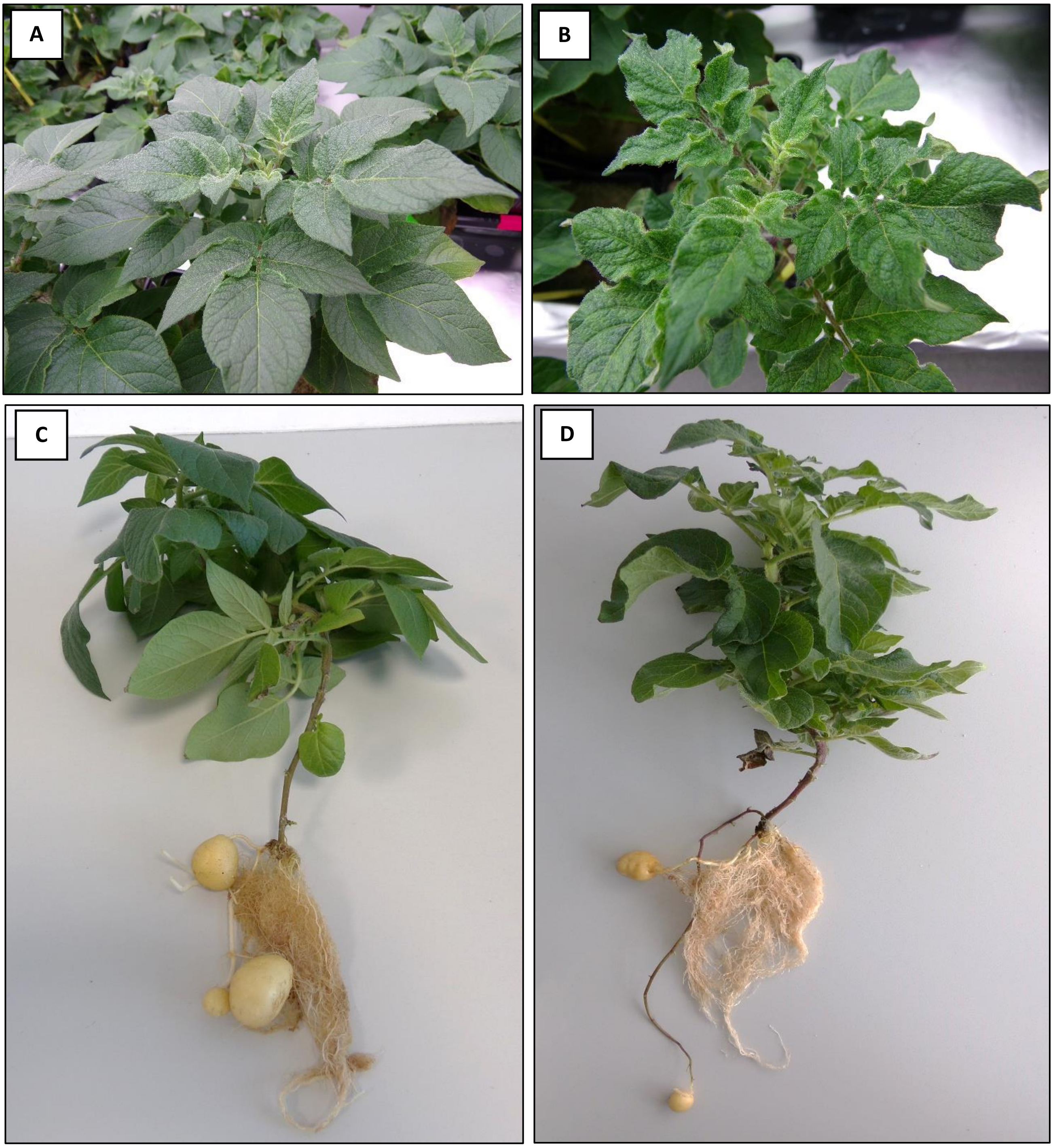

Supplementary Figure 2. Symptoms of persistent PVY infection in potatoes of the Pirol variety after 12 weeks of plant growth in a growth chamber (temperature 18°C, photoperiod 16/8 D/N). Healthy plants are shown in panels A and C, infected by PVY in panels B and D. The virus caused a mosaic pattern on the leaves, as well as crinkling and stunting of the leaves. Leaves and tubers did not show necrotic spots.
