# Supplementary material for: Transcriptomic responses of Solanum tuberosum cv. Pirol to arbuscular mycorrhiza and potato virus Y (PVY) infection
Source: Plant Mol Biol. 2024 Nov 11;114(6):123. doi: 10.1007/s11103-024-01519-9 (PMC11554710; doi:10.1007/s11103-024-01519-9)
Supplement: Supplementary file 4 — Supplementary file4 (DOCX 13 kb) [file 11103_2024_1519_MOESM4_ESM.docx]

Supplementary Table 2. Validation of transcriptomic data via relative expression analysis of StPR-1, StPR-2, and StPT3 in mycorrhizal potato roots.

|  | Relative level of gene expression in root transcriptomes | | | |
| --- | --- | --- | --- | --- |
|  | PVY-negative plants | | PVY-positive plants | |
|  | Control vs. Fm | Control vs. Ri | Control vs. Fm | Control vs. Ri |
| StPR-1 | 86.05 (40.75) | 42.67 (21.56) | 110.64 (38.05) | 22.84 (11.73) |
| StPR-2 | 8.26 (3.80) | 0.34 (0.96) | 4.73 (1.40) | 0.01 (0.41) |
| StPT3 | 65.22 (1124.90) | 172.02 (2505.99) | 115.32 (866.63) | 111. 02 (1291.40) |

This table presents the validation of transcriptomic data by comparing the relative changes in the expression levels of three genes: StPR-1, StPR-2, and StPT3, in the roots of mycorrhizal potatoes. The data were obtained from normalized transcript counts, expressed as FPKM (Fragments Per Kilobase Million). For comparison, values calculated using qPCR are provided in parentheses. In most cases, the results of transcriptomic analysis and qPCR overlap, showing similar trends such as up-regulation or down-regulation of genes. However, the magnitude of these changes often differs between these two methods due to the specific methodologies underlying each technique. qPCR measures gene expression by analyzing total RNA after reverse transcription into cDNA. This approach quantifies the overall abundance of specific transcripts in the sample. In contrast, transcriptomic analysis involves an mRNA enrichment step before sequencing. This enrichment process selectively captures mRNA, potentially leading to differences in the observed expression levels compared to qPCR.
